# Proteome analysis of Norway maple (*Acer platanoides *L.) seeds dormancy breaking and germination: influence of abscisic and gibberellic acids

**DOI:** 10.1186/1471-2229-9-48

**Published:** 2009-05-04

**Authors:** Tomasz A Pawłowski

**Affiliations:** 1 Seed Biochemistry Laboratory, Institute of Dendrology Polish Academy of Sciences, Parkowa 5, 62-035 Kórnik, Poland

## Abstract

**Background:**

Seed dormancy is controlled by the physiological or structural properties of a seed and the external conditions. It is induced as part of the genetic program of seed development and maturation. Seeds with deep physiological embryo dormancy can be stimulated to germinate by a variety of treatments including cold stratification. Hormonal imbalance between germination inhibitors (e.g. abscisic acid) and growth promoters (e.g. gibberellins) is the main cause of seed dormancy breaking. Differences in the status of hormones would affect expression of genes required for germination. Proteomics offers the opportunity to examine simultaneous changes and to classify temporal patterns of protein accumulation occurring during seed dormancy breaking and germination. Analysis of the functions of the identified proteins and the related metabolic pathways, in conjunction with the plant hormones implicated in seed dormancy breaking, would expand our knowledge about this process.

**Results:**

A proteomic approach was used to analyse the mechanism of dormancy breaking in Norway maple seeds caused by cold stratification, and the participation of the abscisic (ABA) and gibberellic (GA) acids. Forty-four proteins showing significant changes were identified by mass spectrometry. Of these, eight spots were identified as water-responsive, 18 spots were ABA- and nine GA-responsive and nine spots were regulated by both hormones. The classification of proteins showed that most of the proteins associated with dormancy breaking in water were involved in protein destination. Most of the ABA- and GA-responsive proteins were involved in protein destination and energy metabolism.

**Conclusion:**

In this study, ABA was found to mostly down-regulate proteins whereas GA up-regulated proteins abundance. Most of the changes were observed at the end of stratification in the germinated seeds. This is the most active period of dormancy breaking when seeds pass from the quiescent state to germination. Seed dormancy breaking involves proteins of various processes but the proteasome proteins, S-adenosylmethionine synthetase, glycine-rich RNA binding protein, ABI3-interacting protein 1, EF-2 and adenosylhomocysteinase are of particular importance. The effect of exogenously applied hormones was not a determining factor for total inhibition (ABA) or stimulation (GA) of Norway maple seed dormancy breaking and germination but proteomic data has proven these hormones play a role.

## Background

Seeds of many plant species enter a period of dormancy when they fail to germinate under favourable conditions. Seed dormancy is controlled by the physiological or structural properties of a seed and external conditions. It is induced as a part of the genetic program of seed development and maturation. Dormancy can be caused by the maternally derived seed covering structures or by embryonic factors, acting individually or in combination [1,2]. In the majority of species with dormancy located in the fully developed mature embryo, dormancy mechanisms appear to be related to reversible metabolic processes (physiological dormancy) [1]. Seeds with deep physiological embryo dormancy can be stimulated to germinate by a variety of treatments including cold stratification, as is the case for Norway maple seeds [3]. Hormonal imbalance (antagonism) between germination inhibitors (e.g. abscisic acid, ABA) and growth promoters (e.g. gibberellin acid, GA) is a main cause of seed dormancy breaking and germination (hormone balance theory of dormancy). Differences in the status of hormones would affect expression of genes required for germination. Using a proteomic approach it is now possible to investigate protein expression during seed dormancy breaking [4-7].

The antagonistic action of the hormones GA and ABA in regulating seed dormancy breaking and germination is well established [8,9]. Hormones act largely in opposition to each other in regulating germination. ABA plays a critical role in the induction and the maintenance of seed dormancy and inhibits the transition from embryonic to germinative growth [10]. Onset and breaking of dormancy depends not only on ABA synthesis but also on sensitivity to ABA [11]. The use of ABA-deficient and ABA-responsive mutants has made a contribution towards understanding the role of ABA in developmental processes, including induction, maintenance and breaking of dormancy [for review see [8,12]]. There is some evidence that genes involved in sensitivity to ABA (*ABI *genes) may be involved in the response to cold during dormancy breakage by affecting the sensitivity of germination to inhibition by ABA [1]. The genes *ABI3*, *ABI4 *and *ABI5 *encode transcription factors that appear to act later in the germination process. Two of these genes, *ABI1 *and *ABI2*, encode protein phosphatases involved in regulating the phosphorylation status of transcription factors. They act as negative regulators of ABA signalling [13].

Gibberellins play a crucial role in promoting seed germination [14-16]. GA has been proposed to function during seed germination in two ways: increasing the growth potential of the embryo and overcoming the mechanical restraint conferred by the seed-covering layers, by weakening the tissues surrounding the radicle. This is further supported by the finding that at least some GA-responsive genes are expressed in non-GA-producing seed tissues [16]. GA-deficient biosynthesis mutants of Arabidopsis (e.g. ga1) and tomato (e.g. gib-1) have been isolated. Seed germination of several of these GA-deficient mutants absolutely depends on the addition of GA to the medium during imbibition [17]. A cold treatment does not stimulate GA biosynthesis directly but rather increases the sensitivity of a seed to GA. The Arabidopsis *GAI *gene, and its orthologues in other species, encode nucleus-localised proteins that act as transcription factors and appear to be negative regulators of the GA-signal transduction pathway. The GAI protein belongs to the DELLA family [8].

The role of gibberellins was examined in germination of Arabidopsis seeds using a proteomic approach [18]. GAs appeared to be involved in controlling the abundance of several proteins associated with germination of Arabidopsis seeds. The cytoskeleton component α-2,4 tubulin appeared to depend on the action of GAs. This is also the case for two isoforms of S-adenosyl-methionine (Ado-Met) synthetase which catalyse the formation of Ado-Met from Met and ATP. Owing to the housekeeping functions of Ado-Met, this event is presumably required for germination and seedling establishment, and might represent a major metabolic control point of seedling establishment. GAs can also play a role in controlling the abundance of β-glucosidase, which might be involved in cell wall loosening in the embryo, needed for cell elongation and radicle extension [18].

The temperate Norway maple tree (*Acer platanoides*) is a model system for investigating broader aspects of physiology, biochemistry and molecular biology of seed dormancy breaking [3]. The seeds are deeply physiologically dormant whatever their moisture level and age. The seeds belong to the category "orthodox", which are seeds tolerant to desiccation, consequently they can be stored for a long time. Their germination must be preceded by moistening and a period of cold stratification at 1–5°C lasting about 3 months. In combination with the availability of genome sequence data, proteomics has opened up enormous possibilities for identifying the total set of expressed proteins as well as expression changes during growth and development [18]. This type of approach brings robust information about the relationship between biological function and physiological changes. In the present study, dormancy breaking and germination of Norway maple seeds and the participation of the two antagonistic hormones ABA and GA in this process were analysed using a proteomic approach.

## Results

### Germination

**Figure 1:**
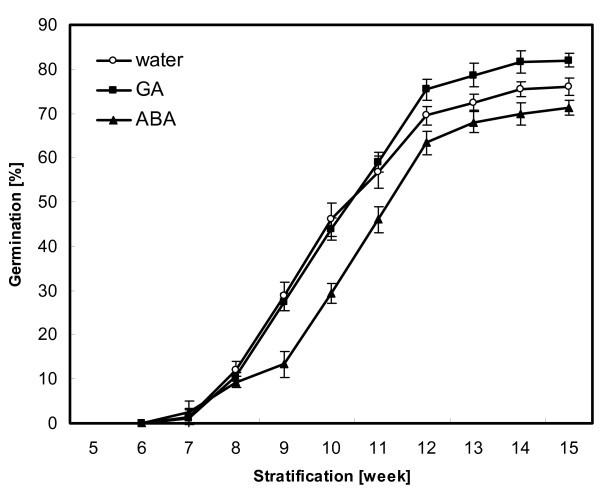
**Germination (at 3°C) of Norway maple seeds after imbibition (in water or in solutions of ABA or GA) and stratification at 3°C**. Error bars represent standard errors, n = 4.

Analysis of Norway maple seed germination after stratification at 3°C (Fig. 1) showed that the application of exogenous ABA (50 μM) had a negative effect on the germination rate (i.e. percentage of germinating seeds). For example, in week 10 of stratification in the presence of ABA the germination rate decreased to 29%, in comparison to 46% in the presence of water. The maximal germination rate for ABA reached 71% in week 15 (76% for water). The application of exogenous GA (100 μM) promoted seed dormancy breaking and increased the final germination rate to 82% (week 15).

### Proteome maps and mass spectrometry results

Qualitative and quantitative changes in proteins were analysed by comparing electrophoregrams at various stages of dormancy breaking (dry dormant seeds, each week of cold stratification at 3°C and germinated seeds). To analyse the ABA- and GA-responsive proteins, significant differences in spot volume between untreated and treated samples were assessed. Protein spots displaying significant up- or down-regulation were regarded as candidates and subject to MS analysis.

**Figure 2:**
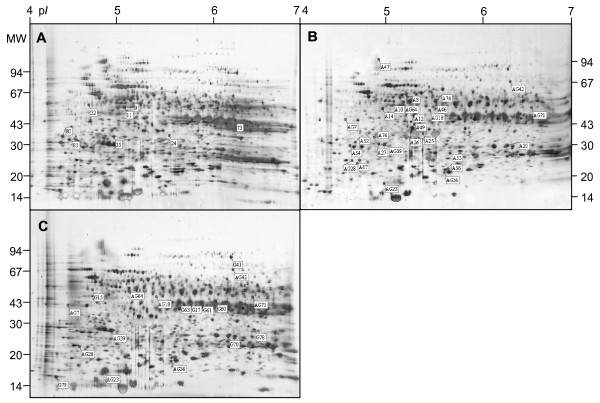
**Positions of the main varying spots on 2D PAGE silver-stained gels of Norway maple seeds during dormancy breaking and germination**. Proteome variation during stratification: (A) in water only, (B) with ABA, (C) with GA. These are the positions of the 44 mapped and identified spots indicated in the master gels (combining the analytical results of 1200 spot groups) by the number that appears in Additional file 1. Specific spots are described as showing variations during stratification in water and between stratification with ABA or GA and stratification in water only.

A total of 1200 protein groups were detected on silver-stained 2D-PAGE using Image Master 5 Platinum. Forty-four spots were significantly variable (ANOVA), representing about 4% of the total spots on the master gels (see Additional file 1 and Fig. 2). The gels from week three of stratification were used to build three master gels, combining the results of statistical analysis of protein volume variation caused by stratification in water, and in ABA and GA solutions. Data were collected from three biological replicates. The first master gel shows the statistically significant proteome variations that occurred throughout cold stratification in water, the second gel shows proteome variation during dormancy breaking in the presence of ABA, the third gel shows proteome variation during dormancy breaking in the presence of GA (Fig. 2A–C panels, respectively).

A total of 18 spots were identified as ABA-responsive proteins and nine spots as GA-responsive proteins (see Additional file 1). Among 18 spots regulated by ABA, eight were up-regulated and 10 down-regulated. The significant influence of ABA on protein variation was observed in week seven (nine spots) and in germinated seeds (eight spots). Among the nine protein spots regulated by GA, seven were up-regulated and two down-regulated. The influence of GA on protein variation was observed especially in week nine (seven spots). It is worth noting that nine spots were regulated by both ABA and GA. Of this group, six spots were down-regulated and three up-regulated by ABA and four spots were down-regulated and five up-regulated by GA. Eight spots were identified as being associated only with seed dormancy breaking in water (four spots in week nine and four in germinated seeds). Two of these spots were up-regulated, six were down-regulated. The 44 proteins showing modulation in expression levels were subjected to amino acid sequence analysis, as described in Methods. The sequence data was compared with protein sequences present in NCBI databases using MASCOT. Sequences were successfully obtained for all of these protein spots.

**Figure 3:**
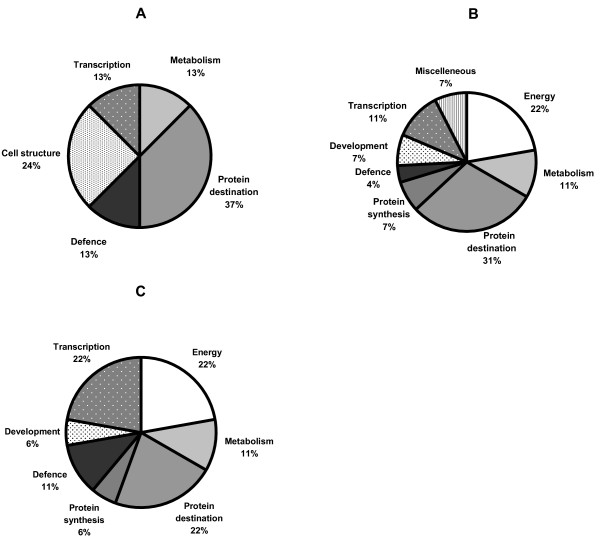
**Assignment of the 44 identified variable protein spots to functional categories using the classification of Bevan *et al***. [19]. (A) Proteins associated with dormancy breaking in water; (B) Proteins regulated by ABA; (C) Proteins regulated by GA.

Identified proteins were classified according to function in the categories described by Bevan *et al*. [19], with some modifications (see Additional file 1). Assignment of the identified proteins to functional categories was also done for dormancy breaking in water, and in the presence of ABA and GA (Fig. 3). Most of the proteins associated with dormancy breaking in water (Fig. 3A) were classified as being involved in protein destination (37% of the proteins; decreased volume in 66% of the proteins of this group was observed). Most of the proteins associated with dormancy breaking in the presence of ABA (Fig. 3B) were also classified as being involved in protein destination (31%; decreased volume in 75% of this group) and energy metabolism (22%; decreased volume in 50% of this group). Most of the proteins associated with dormancy breaking in the presence of GA (Fig. 3C) were classified as being involved in protein destination (22%; increased volume in 75% of this group), energy metabolism (22%; increased volume in all the spots) and transcription (22%; decreased volume in 75% of this group).

The various spots identified as the same protein could correspond either to post translational modification (PTM) of the same protein or to various isoforms.

## Discussion

Although seed germination is a major subject in plant physiological research, there is still a long way to go to elucidate the mechanism involved. Proteomics is now becoming a powerful tool for functional analysis and is being used more and more in studies on seed development, dormancy, dormancy breaking and germination [18,20,21].

Here, a proteomic analysis of seed dormancy breaking and germination in Norway maple was conducted. Most of the changes in protein abundance in Norway maple seeds were observed at the end of stratification and in the germinated seeds. This is the most active period of dormancy breaking when seeds pass from the quiescent state to germination [22]. The effect of exogenously applied hormones was not a determining factor for total inhibition (ABA) or stimulation (GA) of the Norway maple seed dormancy breaking and germination but proteomic data has proven these hormones play a role.

Results from this study correspond with the previous proteomic analysis of European beech (*Fagus sylvatica*) seeds dormancy breaking and germination [4]. Beech seeds were characterised by deep physiological dormancy caused partly by seed coats and partly by endogenous factors in the embryo. Due to their reduced longevity during storage, the seeds are classified in the intermediate category, called also "suborthodox". Seeds in this category can tolerate some desiccation but cannot survive dehydration at 10°C below about 40–50% relative humidity [23]. Norway maple seeds were also characterised by deep physiological dormancy but according to their desiccation tolerance belong to the "orthodox" category and can be stored for a long time without loosing vigour. The changes in abundance of specific proteins including heat shock proteins (HSPs) and Em proteins (up-regulated by ABA), enolase (up-regulated by GA), a proteasome alpha subunit (down-regulated by ABA) and aldolase and NAC (down-regulated by GA), were associated with dormancy breaking and germination of beech as well as Norway maple seeds. The HSPs and LEA proteins isolated from these two species may also play a protective role during water deficit and storage [24].

Proteomic analysis of the seeds of another woody plant, *Prunus campanulata*, provide evidence of the involvement of prunin and dehydrin in the response to warm and cold conditions of stratification, leading to the breaking of dormancy and germination [6]. Prunin refers to globulins of the genus *Prunus*, which comprise the main family of storage proteins synthesised in seeds during embryogenesis. Degradation of prunin which occurred during cold stratification is probably related to GA induction. The reduction in the ABA level during warm or cold stratification coincided with the decrease in the dehydrin level [6]. Two LEA proteins, Lemmi9 and Em (dehydrins also belong to this group), from Norway maple seeds were also up-regulated by ABA and their quantity decreased during dormancy breaking and germination. No storage proteins were identified which could be associated with dormancy breaking.

The mechanisms controlling seed dormancy in Arabidopsis have been characterised by proteomics using the dormant (D) accession [5]. Comparative studies carried out with freshly harvested dormant and after-ripened non-dormant (ND) seeds revealed a specific, differential accumulation of 32 proteins. Exogenous application of ABA to ND seeds strongly impeded germination. This application resulted in an altered accumulation pattern of 71 proteins, with a shift away from accumulation of a major group of proteins, involved mainly in energy and protein metabolism [5]. The same negative effect of ABA was observed on proteins involved in energy metabolism and protein destination during Norway maple seed dormancy breaking. The proteins, HSP, aspartate aminotransferase, EF-2, α-tubulin and LEA, were found in Norway maple as well as Arabidopsis seeds and they are key components controlling seed germination.

Other publications also describe the proteomic investigation of seeds without dormancy. Kim *et al*. [25] identified 16 proteins from germinating rice seeds, notably modulated by either GA or ABA. The examination of two proteins, rice isoflavone resuctase (OsIFR) and rice PR10 (OsPR10), revealed that both are specifically expressed in the embryo and are dramatically down regulated by ABA. Previous investigation of the germination process of rice seeds showed that 148 proteins displayed differently [26]. The down-regulated proteins were mainly storage proteins (e.g. globulin and glutelin), proteins associated with seed maturation (e.g. early embryogenesis protein and late embryogenesis abundant protein) and proteins related to desiccation (e.g. ABA-induced protein and cold-regulated protein). In addition to alpha-amylase, the up-regulated proteins were mainly those involved in glycolysis, such as UDP-glucose dehydrogenase, fructokinase (also up-regulated during dormancy breaking of Norway maple seeds), phosphoglucomutase and pyruvate decarboxylase [26]. A proteomic approach was also used to analyse soybean (*Glycine max*) seed germination [27]. Twenty-four proteins showed changes in abundance. They included nucleotide diphosphate kinase, proglycinin A(1a)B(1b) subunit, thioredoxin fold, 35 ku seed maturation protein, heat shock protein and seed maturation protein PM36.

The function of the proteins identified and the related metabolic pathways involved in Norway maple seed dormancy breaking and germination, in conjunction with the plant hormones implicated in these processes, will be discussed further.

### Protein destination

Classification of the 44 identified Norway maple seed proteins associated with dormancy breaking showed that the majority were involved in protein destination. The study revealed seven HSPs: three were regulated by ABA, two by GA and two were associated with stratification in water. The sequence of four of these proteins corresponded to sequences of HSP70 molecular chaperones. Since HSP70s are considered to be involved with the chaperoning and folding of proteins, these data support the importance of these proteins in maintaining cellular homeostasis and proper protein biogenesis despite the influence of abiotic or biotic factors [28]. Three of the isolated HSP70 proteins were luminal-binding proteins BIP (G61, A14 and G42, DnaK-type molecular chaperones) required for translocation, folding and assembly of secretory and transmembrane proteins passing through the ER secretory pathway. They are known to be actively synthesised during cold-acclimating conditions [28]. The forth HSP70 protein was a copper chaperone (spot 24). Copper (Cu) chaperones constitute a family of small Cu^+^-binding proteins required for Cu homeostasis in eukaryotes [29].

Three destination proteins identified were RuBisCO chaperonins (spots 9, A8 and A10) from the HSP60 family. They bind RuBisCO small and large subunits and are implicated in the assembly of the enzyme oligomer. These proteins show ATPase activity [30].

The number of HSPs identified suggests that they are responsible for maintaining the state of the protein during the very active period of plant development that is seed dormancy breaking and germination.

Three proteasome proteins from Norway maple seeds were isolated: alpha subunit (spot G70, up-regulated by GA), ATPase proteasome subunit P45 (spot AG7, down-regulated by hormones) and 26S proteasome regulatory particle (spotA52, down-regulated by ABA). In plants, the role of proteasomes is associated with regulation of developmental events by controlling the levels of nuclear regulatory proteins (TF) by the ubiquitin-proteasome system in proliferating and developing tissues [31]. Plant responses under the control of the proteasome include: the perception of hormones, photomorphogenesis, trichome development, floral homeosis, environmental adaptation, entrainment of circadian rhythms, disease resistance and senescence [31]. Genetic studies in Arabidopsis have provided evidence for a role of the ubiquitin/26S proteasome pathway in ABA responses, notably during germination [32]. Proteasomes can be involved in seed dormancy breaking by the degradation of transcriptional regulators of diverse metabolic pathways.

Two other proteins isolated from Norway maple seeds were also involved in protein degradation, these proteins were both leucine aminopeptidases (spots: A67, down-regulated by ABA and A46, up-regulated by ABA). Proteolytic enzymes are intricately involved in many aspects of plant physiology and development. They are necessary for protein turnover. Degradation of damaged, mis-folded and potentially harmful proteins provides free amino acids required for the synthesis of new proteins. Furthermore, the selective breakdown of regulatory proteins controls key aspects of plant growth, development, and defence. Proteases are also responsible for the post-translational modification of proteins by limited proteolysis at highly specific sites. Limited proteolysis results in the maturation of enzymes, is necessary for protein assembly and subcellular targeting and controls the activity of enzymes, regulatory proteins and peptides. Proteases are thus involved in all aspects of the plant life cycle ranging from the mobilisation of storage proteins during seed germination to the initiation of cell death and senescence programs [33]. Leucine aminopeptidase, whose accumulation was controlled by ABA, can be the key factor in proteolysis of some regulatory proteins responsible for Norway maple seeds dormancy breaking.

Another interesting protein was serpin (spot 81) whose volume decreased during Norway seed dormancy breaking. The serpins constitute a superfamily of versatile proteins participating in the regulation of complex proteolytic systems. Most serpins are serine proteinase inhibitors of chymotrypsin-like enzymes. Plant serpins are likely to use their irreversible inhibitory mechanism in the inhibition of endogenous and exogenous proteinases capable of breaking down seed storage proteins and in the defence of specific cell types in vegetative tissues [34]. In Norway maple seeds, decreasing abundance of serpin associated with dormancy breaking suggests that proteolysis of storage material essential for germination can begin.

### Energy metabolism

The majority of energy metabolism proteins are involved in glycolysis (spot G60, enolase; spot A20, triosphosphate isomerase), the pentose-phosphate shunt (spot A26, fructose-bisphosphate aldolase), gluconeogenesis (spot A54, fructokinase) or ATP synthesis (spots G15 and AG64, mitochondrial ATPase beta subunits). Two other proteins, namely oxygen evolving proteins (spots A27 and AG39) are associated with the photosystem II complex. They stabilise the manganese cluster, the primary site of water splitting. Dormancy release of seeds is accompanied by ATP accumulation, respiration and other metabolic processes related to energy production [35-37]. ATP is the main energy source for biological processes, including seed germination, and is used in anabolic processes, such as RNA and protein synthesis [35]. The results from this study were similar to the previously reported proteomic investigation of *Fagus sylvatica *seeds [4]. Energy metabolism proteins were also associated with seed dormancy breaking. GA was found to be stimulatory, whereas ABA was inhibitory. This was observed for beech as well as Norway maple seeds.

### Metabolism

The majority of metabolism proteins are involved in amino acid metabolism. After seed imbibition, the content of amino acids increases due to hydrolysis of reserve proteins. The increase in aminotransferase (spot G17) accumulation caused by GA was associated with stratification and germination of Norway maple seeds. Similar stimulation of aspartate aminotransferase activity was observed during germination of peanut [38].

Isovaleryl-CoA dehydrogenase (IVD, spot 73, increased at the beginning of the stratification in water) is an enzyme of the leucine catabolic pathway. In animals, accumulation of IVD mRNA is associated with protein mobilisation, whereas protein sparing results in a decrease of IVD mRNA levels [39]. In plants, protein mobilisation can occur in a number of developmental or nutritional conditions such as germination, senescence or carbohydrate starvation [40]. Germination requires the enzymatic mobilisation of reserves in order to meet the increased metabolic demands of growth. Increasing levels of isovaleryl-CoA dehydrogenase during stratification of Norway maple seeds confirms such a mechanism.

Adenosylhomocysteine is a competitive inhibitor of S-adenosylmethionine (SAM)-dependent methyl transferase reactions. Therefore, adenosylhomocysteinase (spot A74, increased during stratification in water, down-regulated by ABA) may play a key role in the control of DNA or of other substrates methylation via regulation of the intracellular concentration of adenosylhomocysteine. The *hog1 *mutant of Arabidopsis showed reduced adenosylhomocysteinase activity destabilising maintenance methylation and affecting expression of thousands of genes. The *hog1 *mutant plants grow slowly and have low fertility and reduced seed germination. Complementation of the *hog1 *mutation with a T-DNA containing the gene coding for adenosylhomocysteinase restored DNA methylation, fast growth, and normal seed viability [41]. It seems likely that adenosylhomocysteinase acts via regulation of DNA methylation which affects expression of certain genes responsible for seed dormancy breaking.

Adenosylhomocysteinase is also part of the methionine synthesis pathway, along with S-adenosylmethionine (SAM) synthetase (spot AG71, down-regulated by ABA and up-regulated by GA). (SAM) synthetase catalyses from methionine and ATP the formation of the SAM, which is also required for the maintenance and recycling of methylation in plants. The SAM synthetase is a fundamental component controlling metabolism in the transition from a quiescent to a highly active state during Arabidopsis seed germination [42]. Its strong accumulation was observed at the radicle emergence step. The inhibitory effect of ABA on Norway maple seed dormancy breaking can be associated with decreasing of abundance of SAM synthetase.

SAM can be metabolised via two pathways, leading to ethylene and polyamine synthesis. Ethylene is biosynthesised via the following pathway: methionine – SAM – 1-aminocyclopropane 1-carboxylic acid (ACC) – ethylene. Ethylene is produced by all higher plants and regulates many aspects of growth and development, ranging from seed germination to flower fading, fruit ripening and leaf senescence [9,43]. Ethylene promotes seed dormancy breaking and germination and counteracts ABA effects. Results from this study show that exogenously applied ABA down-regulates SAM synthetase and it can be proposed that via a negative effect on ethylene synthesis Norway maple seeds dormancy breaking is inhibited.

Polyamines are found ubiquitously in higher plants and it has been proposed that they play an important role in the regulation of plant growth and development. More specifically they are important in induction of protein synthesis, DNA replication, cell division and the response to abiotic stress. Their activity is associated with seed dormancy breaking and germination [44,45]. Alterations in the level of endogenous polyamines and the accelerating influence of exogenous polyamines was observed on dormancy breaking and germination of Norway maple seeds during stratification [46]. Aminoaldehyde dehydrogenase (spot A76, down-regulated by ABA) is associated with metabolism of polyamine oxidation products in plants. The polyamines are metabolised finally by an aminoaldehyde dehydrogenase to β-alanine and γ-aminobutyric acid (GABA) [47]. GABA is a non-protein amino acid that might function as an intercellular signalling molecule. Environmental stresses or transient environmental factors increase GABA accumulation [48]. GABA might play some role in seed dormancy breaking but this hypothesis needs to be verified experimentally.

### Transcription

A nascent polypeptide-associated complex (NAC, spot AG28) was down-regulated by GA and ABA. It has been proposed to protect the nascent chains from premature interaction with other cellular proteins, associate with DNA junctions and play a role in other processes including transcription regulation and mitochondrial protein import. The NAC alpha subunit contains an ubiquitin-associated domain, which is found in several proteins involved in the ubiquitin-proteasome pathway for protein degradation. NAC also interacts with HSP70 [49].

Another protein associated with transcription is the DEAD box RNA helicase (spot 11, down-regulated during stratification in water). Helicases are ubiquitous enzymes that catalyse the unwinding of energetically stable duplex DNA (DNA helicases) or duplex RNA secondary structures (RNA helicases). Most helicases are members of the DEAD-box protein superfamily and play essential roles in basic cellular processes such as DNA replication, repair, recombination, transcription, ribosome biogenesis and translation initiation [50]. Therefore, helicases might be playing an important role in regulating plant growth and development. DEAD-box helicases are very sensitive to the abiotic stresses that reduce plant growth and productivity. ABA treatment induced DEAD-box helicase mRNA in the roots, indicating a role for ABA-dependent pathways in abiotic stress. DEAD box RNA helicase can be involved in maintenance of dormancy of Norway maple seeds. Stratification decreased its accumulation and abolished the inhibitory effect on germination.

Glycine-rich RNA binding proteins (spots G79 and AG36) were down-regulated by GA and up-regulated by ABA. They have been implicated in post-transcriptional regulation of gene expression in plants under various stress conditions. The expression of an ABA-responsive glycine-rich protein correlates with the level of seed dormancy in beech (*Fagus sylvatica*) seeds [51]. Mortensen *et al*. [52] suggested that the degree of dormancy of beech seeds is associated with the expression of the ABA-responsive cDNA that is a clone of the dormancy-related gene *GRPF1*, encoding a glycine-rich RNA-binding protein. In Norway maple seeds this protein might also be associated with dormancy breaking, but further investigations are required to address this.

ABI3-interacting protein 1 (CnAIP1, spot AG18, down-regulated by ABA and up-regulated by GA in week three of stratification) is associated with transcription factor ABA-insensitive 3 protein (ABI3), which is a central regulator of plant seed development and ABA signalling. ABI3 determines ABA sensitivity and plays a central role in establishing desiccation tolerance and dormancy during zygotic embryogenesis. ABI3 proteins are abundant in mature seeds, but disappear after germination. They activate embryo maturation pathways and simultaneously repress germination. Dormancy breaking reduced expression of ABI3 and the level of ABI3 can be up-regulated by ABA. ABI3 might function as a general regulator imprinting the timing of developmental transitions [53]. It is a key determinant of seed-specific expression, for example, it controls genes encoding Em protein [54], peroxiredoxin [55] and HSP [56] (proteins found also in stratified Norway maple seeds). Proteins which interact with ABI3 play a role in activation of transcription. These interactions prevent the premature activation of genes associated with germination and growth. Jones *et al*. [57] reported three proteins from *Avena fatua *seeds: AfVIP1 (homolog of CnAIP1), 2 and 3, which interact with ABI3. AfVIP1 and 3 may play specific roles in transition of seeds to germination. Arabidopsis ABI3-interacting proteins (AIPs, homologues of AfVIPs) show homology to existing transcription factors and may function with ABI3 [58]. AIP1 protein shows high homology to the plant transcription factor CONSTANS (CO), flowering time regulatory protein. The function of CO appears to be the repression of ABI3 [58]. On the basis of these results it can be concluded that AIP1 may be required together with ABI3 during Norway maple seed development. AIP2 is known to be negatively regulated in ABA signalling by targeting ABI3 for post-translational regulation by 26S proteasomes (confirmed by this study) [10]. Probably in the same manner, the function of AIP1 protein from Norway maple seeds leads to removal of the negative effect of ABA on seed dormancy breaking and germination.

### Plant defence

Some protein spots were identified that were involved in plant defence and linked to oxidative stress: peroxiredoxin (PRX, spots G78 and 80), glutathione S-transferase (A55) and peroxidase (G63). PRXs are antioxidant proteins which confer a protective role in cells through peroxidase activity by reducing hydrogen peroxide, peroxynitrite and organic hydroperoxides. They play a putative role in protecting seeds from desiccation damage by exposure to reactive oxygen species (ROS). Peroxiredoxin can sense harsh environmental surroundings and play a part in the inhibition of germination under unfavourable conditions [55]. It has been suggested that peroxiredoxins play a role in dormancy [55], however, it is now thought only via a relationship between expression level and dormancy maintenance rather than the establishment of dormancy *per se *[59]. Glutathione S-transferase is involved in conjugation of reduced glutathione to a wide number of exogenous and endogenous hydrophobic electrophiles. Peroxidase (spot G63) is a homolog of Euphorbia latex peroxidase, a calmodulin (CaM)-binding protein activated by the Ca^2+^/CaM system. Peroxidase might be another node in the Ca^2+^/H_2_O_2_-mediated plant defence system, having both positive and negative effects in regulating H_2_O_2 _homeostasis [60].

A strong, negative correlation was found between germination capacity and ROS, such as superoxide radical and hydrogen peroxide, as well as with lipid hydroxyperoxides [61]. Germination of cereals was accompanied by extensive changes in the redox state of seed proteins. Proteins present in an oxidised form in dry seeds were converted to the reduced state following imbibition [62].

Recent studies have indicated that protein oxidation is not necessarily a deleterious phenomenon in plants. ROS have been invoked to play a role in cellular signalling (for review see [63]) raising the hypothesis that these compounds can facilitate the shift from a dormant to a non-dormant status in seeds. After-ripening of dormant sunflower (*Helianthus annuus *L.) seeds entailed a progressive accumulation of ROS, namely superoxide anions and hydrogen peroxide, in cells of embryonic axes. This accumulation occurred concomitantly with lipid peroxidation and oxidation (carbonylation) of specific embryo proteins. It has been proposed that the mechanism for seed dormancy alleviation involves ROS production and targeted changes in protein carbonylation patterns [64].

ROS-scavenging enzymes, present in Norway maple seeds, firstly can play a protective role against stress and secondly can play a role in seed dormancy breaking through changing the level of ROS.

### Protein synthesis

Some protein spots were identified that were involved in protein synthesis. These were represented by elongation factor 2 (EF-2, spot A12, increased during stratification in water and down-regulated by ABA; spot G41, up-regulated by GA) and ribosomal protein PO (spot A25, increased during stratification in water and down-regulated by ABA). EF-2 promotes the GTP-dependent translocation of the nascent protein chain from the A-site to the P-site of the ribosome. A high-level expression of EF is a prerequisite for maintaining rapid protein synthesis and cell division in meristematic tissues, which is necessary for root elongation [65]. Twardowski and Szczotka [65] reported the changes of EF1 activity crucial for protein biosynthesis during dormancy breaking of Norway maple seeds. They suggested that polyamines can be regulators of the translation process by modulating the activity of EF1. Authors also observed the increase in the level of protein synthesis and in ribosome accumulation associated with Norway maple seeds dormancy breaking [22,65]. These results show that elongation factors can play an important role in the mechanism of seed dormancy breaking, where they are responsible for protein synthesis and cell division in the root meristem.

### Development

Lemmi9 (spot AG23) was up-regulated by ABA and down-regulated by GA. It belongs to the late embryogenesis abundant (LEA) protein family. LEA proteins are produced in many plant organs during plant development and under stress conditions [66]. In seeds, LEA proteins are related to the acquisition of desiccation tolerance during development and their expression is regulated by ABA [67]. Some of the closer studied LEA proteins which appear during seed dormancy breaking are dehydrins [5,6], detected also in seeds of Norway maple [68]. LEA protein levels declined simultaneously with germination [69]. Analysis of gene expression associated with seed dormancy breaking in wild oat (*Avena fatua*) [70] showed that cDNA clones encoding LEA proteins were regulated by ABA and GA. GA treatment of dormant seeds breaks dormancy and lowers transcript levels of LEA, whereas ABA treatment increases transcript levels of LEA. Lemmi9 displays ethylene-regulated expression in response to drought, ABA and wounding [71]. Ethylene is involved in regulating the interconnected molecular processes that control dormancy release and germination [8].

Another spot identified as LEA protein, in fact represents the early methionine-labeled (Em) protein (spot A56, up-regulated by ABA). The Em proteins correspond to the class I LEA proteins and are essentially seed specific [72]. The expression of an Em gene is activated by ABI3 protein in the presence of ABA. Baumbusch *et al*. [73] found that the abundances of two genes encoding the Em protein (AtEm1 and AtEm6) are affected by imbibition and the cold temperature used for *Arabidopsis *seed dormancy breaking.

### Cell structure

The expression of the alpha- and beta-tubulins (spots 32 and 35, respectively) decreased during stratification in water. From previous research it is known that changes in accumulation of beta-tubulin are associated with Norway maple seed dormancy breaking and germination [74]. Chibani *et al*. [5] observed similar results during germination of dormant Arabidopsis seeds. Taken together, the expression of alpha- and beta-tubulins is the important determinant of completion of dormancy breaking and initiation of germination and growth.

## Conclusion

Temperate Norway maple (*Acer platanoides*) tree is a model system for investigation of broader aspects of physiology, biochemistry and molecular biology of seeds dormancy breaking and germination. The seeds are deeply physiologically dormant whatever their moisture level and age. They belong to the category "orthodox" as the seeds are tolerant to desiccation. Their germination must be preceded by moistening and a period of cold stratification at 1–5°C lasting about three months. A proteomic approach was used to analyse the mechanism of dormancy breaking in Norway maple seeds caused by cold stratification and the participation of the abscisic (ABA) and gibberellic (GA) acids. Forty-four proteins showing significant changes in expression levels were identified by mass spectrometry. The inhibitory effect of ABA on dormancy breaking can be due to the proteins which were down-regulated: fructose-biphosphate aldolase, fructokinase, ATPase, SAM synthetase, HSPs and proteasome proteins. Proteins up-regulated by ABA: oxygen-evolving protein, adenosylhomocysteinase, ABI3-interacting protein 1 and glycine-rich RNA binding protein can be associated with inhibition of germination. The role of GA in promoting seed germination can be performed by up-regulated proteins: ATPase, EF-2, aminotransferase and proteasome proteins. Glycine-rich RNA binding protein, down-regulated by GA, can also be associated with regulation of seed dormancy breaking. The proteins which can be associated with promotion of the germination and are not regulated by these hormones are: isovaleryl-CoA-dehydrogenase and copper chaperone. DEAD box RNA helicase, alpha and beta-tubulins, peroxiredoxin and serpin can be associated with inhibition of germination and are not regulated by the hormones investigated.

## Methods

### Plant materials and experimental design

Norway maple (*Acer platanoides *L.) seeds were collected in the Kórnik Arboretum (Poland) in the autumn of 2005. Initially, the seeds were dried at ambient temperature and humidity until they reached a moisture content of 10% (fresh weight basis). They were then stored in plastic containers at -3°C. Prior to the experiments, they were imbibed for 48 h at room temperature in water or aqueous solutions of ABA (50 μM) or GA (100 μM). These concentrations were the most efficient for inhibition (ABA) or stimulation (GA) of Norway maple germination as was investigated previously (data not published). The seeds were then subject to cold stratification at 3°C (i.e. a temperature that breaks their dormancy) for up to 15 weeks in closed plastic trays without medium and in the dark. The germination test (four replicates of 50 seeds each) was carried out at 3°C in accordance with the recommendations of the International Seed Testing Association [75].

### Protein extraction

Seed samples were taken each week during cold stratification with water, ABA or GA at 3°C, from dry dormant seeds to germinated seeds (with 1 mm protruded radicle). Extracts for electrophoresis were prepared as recommended by Bergervoet *et al*. [76]. For each extract, embryo axes of 15 seeds were homogenised in 0.5 mL of 10% (w/v) solution of TCA in acetone containing 0.07% (v/v) β-mercaptoethanol. After protein precipitation for 45 min at -20°C, the homogenate was centrifuged at 16 000 × *g *for 5 min at 4°C. The pellet was resuspended in 1 mL of acetone containing 0.07% (v/v) β-mercaptoethanol and centrifuged again at 16 000 × *g *for 20 min at 4°C. After the supernatant was discarded, the pellet was dried in a vacuum. The proteins were dissolved in lysis buffer, containing 9 M urea, 0.5% (w/v) CHAPS, 2% (v/v) β mercaptoethanol and 2% (v/v) 2-D Pharmalyte 4–7. After centrifugation, the total protein concentration was measured as described by Ramagli and Rodriguez [77] and adjusted to 1 μg/μL.

### Protein electrophoresis

Proteins were separated using a horizontal 2D PAGE system (Multiphor II, GE Healthcare, Little Chalfont, UK). All separations were performed at 15°C on precast gels. In the first dimension, a precise Immobiline DryStrip gel (GE Healthcare) was used with a linear pH gradient from 4 to 7. Each gel was loaded with 25 μg (for silver staining) or 100 μg protein (for colloidal Coomassie Blue). The Immobiline DryStrip gels were equilibrated twice for 10 min each. In the first equilibration step, 0.25% (w/v) DTT was added to the equilibration buffer containing 50 mM Tris/HCl (pH 6.8), 6 M urea, 30% (w/v) glycerol and 2% (w/v) SDS. In the second equilibration step, 4.5% (w/v) iodoacetamide was added to the equilibration buffer instead of DTT. In the second dimension, a precast SDS-PAGE Excel Gradient 8–18 gel (GE Healthcare) was used. A mixture of molecular weight markers (GE Healthcare) was loaded next to the gel. After electrophoresis, the gels were silver stained [78] for densitometric analyses or stained with colloidal Coomassie Blue [79] for the MS analyses.

### Analysis of 2D PAGE gels

The gels were scanned and evaluated using 2D Image Master 5 Platinum software (GE Healthcare). After spot detection, 2D gels (three from three independent biological samples) were aligned and matched, and the normalised spot volumes determined quantitatively. The groups were detected afterwards. For each matched spot, the % volume was calculated as the volume divided by the total volume of matched spots. Statistics were carried out on the groups (%vol) including, the mean (100%) and the mean squared deviation (MSD). The spots showing the greatest variations were subject to ANOVA and Tukey-Kramer HSD testing (JMP software, SAS Institute, Cary, USA) in order to retain spots for which the two factors – stratification time (week) and variant (water, ABA and GA) – had a significant effect (*p *< 0.05) on the % volume of each spot. The significantly variable proteins were identified by ESI-MS/MS.

### Mass Spectrometry (MS)

Peptide mixtures were analysed by LC coupled to an LTQ-FTICR mass spectrometer (Hybrid-2D-Linear Quadrupole ITFT-ICR Mass Spectrometer, Thermo Electron, San Jose, CA). Prior to analysis, gel slices were subject to a standard "in-gel digestion" procedure, according to Candiano *et al*. [79].

Acquired raw data were processed by MASCOT Search (Matrix Science, London, UK, locally installed ) against the NCBI nonredundant database. The respective search parameters for precursor and product ion mass tolerance were ± 40 ppm and ± 0.8 Da, with allowance made for one missed semiTrypsin, fixed modifications of cysteine through carbamidomethylation and variable modification through lysine carbamidomethylation and methionine oxidation.

## Authors' contributions

TAP conceived the study and performed the experiments, analysed the images, statistical and MS data and wrote the manuscript. Author read and approved the final manuscript.

## Supplementary Material

Additional file 1 **Table 1**. Functional classification of Norway maple seed proteins whose level varied significantly during dormancy breaking in water, with or without ABA or GA. Click here for file
