# Supplementary material for: Proteome analysis of Norway maple (Acer platanoides L.) seeds dormancy breaking and germination: influence of abscisic and gibberellic acids
Source: BMC Plant Biol. 2009 May 4;9:48. doi: 10.1186/1471-2229-9-48 (PMC2688491; doi:10.1186/1471-2229-9-48)
Supplement: Additional file 1 — Table 1. Functional classification of Norway maple seed proteins whose level varied significantly during dormancy breaking in water, with or without ABA or GA. [file 1471-2229-9-48-S1.doc]

| **Table 1 -** Functional classification of Norway maple seed proteins whose level varied significantly during dormancy breaking in water, with or without ABA or GA. | | | | | | | | | | | | | | | | | | | | | |
| --- | --- | --- | --- | --- | --- | --- | --- | --- | --- | --- | --- | --- | --- | --- | --- | --- | --- | --- | --- | --- | --- |
| Spot no.*^a^* | Exp. *M*r/p*I* | Theo. *M*r/p*I* | Protein ID | Organism accession no. | % cov.*^b^* | Peptides*^c^* | Water | | | | | Mean % volume^d^  ABA | | | | | GA | | | | |
|  |  |  |  |  |  |  | dry | 3 | 7 | 9 | g | | 3 | 7 | 9 | g | | 3 | 7 | 9 | g |
| Protein destination | | |  |  |  |  |  | | | | | | | | | | | | | | |
| 9 | 65/5.0 | 63/5.9 | RuBisCO chaperonin | [*Pisum sativum* 2506277](http://212.87.29.243/mascot/cgi/protein_view.pl?file=../data/20061220/F029088.dat&hit=gi|2506277&px=1&protscore=425.253333333333&_mudpit=1&_ignoreionsscorebelow=50) | 26 | 12 | 0.18 | 0.13 | 0.12 | 0.00 | 0.07 | | 0.13 | 0.04 | 0.12 | 0.12 | | 0.07 | 0.12 | 0.06 | 0.06 |
| A8 | 67/5.0 | 63/5.9 | RuBisCO chaperonin | [*P. sativum* 2506277](http://212.87.29.243/mascot/cgi/protein_view.pl?file=../data/20061220/F029072.dat&hit=gi|2506277&px=1&protscore=293.11&_mudpit=1&_ignoreionsscorebelow=50) | 13 | 7 | 0.08 | 0.05 | 0.11 | 0.19 | 0.02 | | 0.05 | 0.05 | 0.07 | 0.04 | | 0.07 | 0.06 | 0.01 | 0.04 |
| A10 | 62/4.9 | 63/5.8 | RuBisCO chaperonin | [*P. sativum* 2506277](http://212.87.29.243/mascot/cgi/protein_view.pl?file=../data/20061220/F029083.dat&hit=gi|2506277&px=1&protscore=249.773333333333&_mudpit=1&_ignoreionsscorebelow=50) | 15 | 6 | 0.09 | 0.06 | 0.07 | 0.08 | 0.12 | | 0.06 | 0.07 | 0.07 | 0.09 | | 0.00 | 0.11 | 0.10 | 0.08 |
| 24 | 36/5.4 | 12/4.9 | Copper chaperone | *Plantago major* 53748477 | 11 | 2 | 0.36 | 0.74 | 0.16 | 0.02 | 0.06 | | 0.62 | 0.10 | 0.10 | 0.05 | | 0.25 | 0.32 | 0.18 | 0.03 |
| G61 | 47/6.0 | 74/5.1 | Luminal-binding protein 5 (BiP 5) | [*Nicotiana tabacum* 729623](http://212.87.29.243/mascot/cgi/protein_view.pl?file=../data/20061220/F029076.dat&hit=gi|729623&px=1&protscore=240.98&_mudpit=1&_ignoreionsscorebelow=50) | 10 | 5 | 0.55 | 0.51 | 0.27 | 0.03 | 0.05 | | 0.49 | 0.41 | 0.32 | 0.31 | | 0.43 | 0.16 | 0.51 | 0.13 |
| A14 | 53/4.8 | 73/5.1 | Luminal-binding protein (BiP) | [*Solanum lycopersicum* 1346172](http://212.87.29.243/mascot/cgi/protein_view.pl?file=../data/20061220/F029084.dat&hit=gi|1346172&px=1&protscore=321.205714285714&_mudpit=1&_ignoreionsscorebelow=50) | 13 | 7 | 0.10 | 0.14 | 0.17 | 0.13 | 0.05 | | 0.10 | 0.23 | 0.09 | 0.16 | | 0.07 | 0.19 | 0.10 | 0.04 |
| AG42 | 79/6.4 | 71/5.2 | Heat shock protein 70 | [*Zea mays* 123593](http://212.87.29.243/mascot/cgi/protein_view.pl?file=../data/20060724/F023216.dat&hit=gi|123593&px=1&protscore=281.607142857143&_mudpit=1&_ignoreionsscorebelow=35) | 14 | 6 | 0.05 | 0.04 | 0.08 | 0.05 | 0.04 | | 0.04 | 0.06 | 0.04 | 0.04 | | 0.09 | 0.17 | 0.05 | 0.05 |
| A52 | 32/4.5 | 31/5.0 | 26S proteasome regulatory particle | [*Camellia sinensis* 113196591](http://212.87.29.243/mascot/cgi/protein_view.pl?file=../data/20061220/F029085.dat&hit=gi|113196591&px=1&protscore=243.93&_mudpit=1&_ignoreionsscorebelow=50) | 19 | 6 | 0.07 | 0.02 | 0.14 | 0.05 | 0.07 | | 0.06 | 0.08 | 0.07 | 0.09 | | 0.06 | 0.12 | 0.03 | 0.06 |
| AG7 | 44/4.4 | 48/5.0 | ATPase proteasome subunit P45 | [*Medicago truncatula* 92871663](http://212.87.29.243/mascot/cgi/protein_view.pl?file=../data/20061220/F029084.dat&hit=gi|92871663&px=1&protscore=1534.25297297297&_mudpit=1&_ignoreionsscorebelow=50) | 62 | 38 | 0.00 | 0.00 | 0.02 | 0.07 | 0.07 | | 0.00 | 0.00 | 0.00 | 0.03 | | 0.00 | 0.02 | 0.00 | 0.05 |
| G70 | 29/6.3 | 27/5.9 | Proteasome subunit alpha | [*N. tabacum* 12229948](http://212.87.29.243/mascot/cgi/protein_view.pl?file=../data/20061220/F029075.dat&hit=gi|12229948&px=1&protscore=320.84&_mudpit=1&_ignoreionsscorebelow=50) | 26 | 10 | 0.10 | 0.14 | 0.08 | 0.10 | 0.05 | | 0.12 | 0.00 | 0.04 | 0.04 | | 0.09 | 0.13 | 0.16 | 0.07 |
| A46 | 60/5.4 | 62/8.3 | Leucine aminopeptidase 2 | [*Oryza sativa* 75261364](http://212.87.29.243/mascot/cgi/protein_view.pl?file=../data/20061220/F029081.dat&hit=gi|75261364&px=1&protscore=357.17&_mudpit=1&_ignoreionsscorebelow=50) | 13 | 10 | 0.16 | 0.15 | 0.10 | 0.08 | 0.15 | | 0.20 | 0.15 | 0.11 | 0.22 | | 0.18 | 0.13 | 0.09 | 0.20 |
| A67 | 24/4.6 | 31/5.3 | Leucine aminopeptidase | [*Petroselinum crispum* 1483563](http://proteom.pl/mascot/cgi/protein_view.pl?file=../data/20060928/F026146.dat&hit=gi|1483563&px=1&protscore=173.83&_mudpit=1&_ignoreionsscorebelow=40) | 15 | 5 | 0.09 | 0.04 | 0.16 | 0.10 | 0.02 | | 0.04 | 0.02 | 0.03 | 0.05 | | 0.06 | 0.08 | 0.06 | 0.04 |
|  |  |  |  |  |  |  |  |  |  |  |  |  |  |  |  |  |  |  |  |  |  |
| 81 | 34/4.4 | 43/5.4 | Serpin | [*Triticum aestivum* 1885350](http://212.87.29.243/mascot/cgi/protein_view.pl?file=../data/20061220/F029089.dat&hit=gi|1885350&px=1&protscore=345.624285714286&_mudpit=1&_ignoreionsscorebelow=50) | 22 | 7 | 0.12 | 0.11 | 0.09 | 0.08 | 0.08 | | 0.10 | 0.12 | 0.07 | 0.12 | | 0.07 | 0.11 | 0.06 | 0.07 |
| Energy | | |  |  |  |  |  |  |  |  |  | |  |  |  |  | |  |  |  |  |
| A20 | 30/6.3 | 28/5.9 | Triosphosphate isomerase | *Solanum tuberosum* 76573375 | 30 | 8 | 0.11 | 0.15 | 0.15 | 0.14 | 0.08 | | 0.19 | 0.29 | 0.20 | 0.14 | | 0.10 | 0.09 | 0.13 | 0.17 |
| A26 | 36/5.1 | 39/6.0 | Fructose-bisphosphate aldolase | *A. thaliana* 15231715 | 8 | 2 | 0.18 | 0.29 | 0.08 | 0.19 | 0.05 | | 0.29 | 0.10 | 0.10 | 0.08 | | 0.21 | 0.18 | 0.14 | 0.23 |
| A54 | 31/4.5 | 34/5.5 | Fructokinase | *S. tuberosum* 585973 | 9 | 2 | 0.06 | 0.05 | 0.13 | 0.00 | 0.15 | | 0.05 | 0.04 | 0.05 | 0.14 | | 0.00 | 0.09 | 0.04 | 0.13 |
| G60 | 45/6.2 | 48/5.9 | Enolase 2 | *Hevea brasiliensis* 14423687 | 31 | 15 | 0.36 | 0.23 | 0.10 | 0.06 | 0.04 | | 0.20 | 0.10 | 0.14 | 0.13 | | 0.07 | 0.11 | 0.20 | 0.11 |
| G15 | 60/4.7 | 60/5.8 | Mitochondrial ATPase beta subunit | *Nicotiana sylvestris* 3676294 | 6 | 2 | 0.06 | 0.03 | 0.09 | 0.00 | 0.05 | | 0.07 | 0.09 | 0.02 | 0.04 | | 0.07 | 0.10 | 0.03 | 0.04 |
| AG64 | 61/5.0 | 60/5.9 | ATPase beta subunit, mitochondrial precursor | *H. brasiliensis* 231586 | 40 | 23 | 0.10 | 0.15 | 0.08 | 0.08 | 0.12 | | 0.09 | 0.01 | 0.03 | 0.11 | | 0.06 | 0.08 | 0.11 | 0.13 |
| A27 | 32/4.8 | 35/5.7 | Oxygen-evolving protein 33 kDa | *A. thaliana* 22571 | 10 | 2 | 0.68 | 0.18 | 0.48 | 0.17 | 0.32 | | 0.24 | 0.29 | 0.56 | 0.42 | | 0.29 | 0.32 | 0.55 | 0.29 |
| AG39 | 32/4.8 | 36/5.8 | Oxygen-evolving protein 1 | *S. tuberosum* 131385 | 19 | 6 | 0.68 | 0.28 | 0.51 | 0.21 | 0.22 | | 0.28 | 0.39 | 0.49 | 0.49 | | 0.53 | 0.35 | 0.43 | 0.39 |
| Metabolism | | |  |  |  |  |  | | | | | | | | | | | | | | |
| AG71 | 49/6.6 | 43/5.8 | S-adenosylmethionine synthetase 3 | *S. lycopersicon* 1170939 | 43 | 23 | 0.03 | 0.05 | 0.10 | 0.03 | 0.19 | | 0.07 | 0.07 | 0.04 | 0.10 | | 0.05 | 0.06 | 0.11 | 0.17 |
| G17 | 48/5.8 | 50/6.9 | Aminotransferase | *M. truncatula* 92877835 | 7 | 3 | 0.64 | 0.65 | 0.26 | 0.15 | 0.23 | | 0.66 | 0.20 | 0.41 | 0.46 | | 0.32 | 0.29 | 0.53 | 0.31 |
| 73 | 44/6.3 | 45/7.5 | Isovaleryl-CoA-dehydrogenase | *A. thaliana* 5596622 | 9 | 3 | 0.08 | 0.32 | 0.00 | 0.15 | 0.06 | | 0.20 | 0.11 | 0.00 | 0.00 | | 0.00 | 0.08 | 0.16 | 0.06 |
| A74 | 63/5.4 | 54/5.6 | Adenosylhomocysteinase | *Catharanthus roseus* 407412 | 13 | 5 | 0.07 | 0.13 | 0.12 | 0.00 | 0.05 | | 0.05 | 0.00 | 0.02 | 0.09 | | 0.04 | 0.08 | 0.10 | 0.08 |
| A76 | 35/4.7 | 55/5.5 | Aminoaldehyde dehydrogenase | *Pisum sativum* 15131692 | 11 | 5 | 0.10 | 0.10 | 0.10 | 0.06 | 0.05 | | 0.04 | 0.04 | 0.04 | 0.08 | | 0.02 | 0.06 | 0.06 | 0.22 |
| Transcription | | |  |  |  |  |  | | | | | | | | | | | | | | |
| AG28 | 25/4.5 | 24/4.4 | Nascent polypeptide-associated complex | *M. truncatula* 92878643 | 14 | 2 | 0.37 | 0.33 | 0.31 | 0.34 | 0.16 | | 0.21 | 0.21 | 0.17 | 0.48 | | 0.27 | 0.26 | 0.17 | 0.38 |
| 11 | 57/4.9 | 47/5.4 | DEAD box RNA helicase | *P. sativum* 25809054 | 38 | 24 | 0.24 | 0.20 | 0.06 | 0.02 | 0.07 | | 0.10 | 0.06 | 0.05 | 0.15 | | 0.10 | 0.10 | 0.04 | 0.09 |
| AG18 | 50/5.3 | 32/9.3 | ABI3-interacting protein 1 (CnAIP1) | *Chamaecyparis nootkatensis* 30421186 | 3 | 1 | 0.09 | 0.04 | 0.09 | 0.05 | 0.02 | | 0.03 | 0.08 | 0.05 | 0.09 | | 0.09 | 0.10 | 0.02 | 0.07 |
| G79 | 15/4.3 | 17/5.5 | Glycine-rich RNA binding protein 2 | *Pelargonium x hortorum* 2267567 | 14 | 2 | 0.25 | 0.22 | 0.20 | 0.50 | 0.36 | | 0.23 | 0.19 | 0.19 | 0.35 | | 0.06 | 0.18 | 0.18 | 0.47 |
| AG36 | 21/5.5 | 7/7.9 | Glycine-rich RNA binding protein | *Citrus unshiu* 7024451 | 29 | 8 | 0.11 | 0.07 | 0.18 | 0.15 | 0.03 | | 0.02 | 0.06 | 0.10 | 0.18 | | 0.21 | 0.15 | 0.06 | 0.11 |
| Defense | | |  |  |  |  |  | | | | | | | | | | | | | | |
| G78 | 32/6.6 | 18/5.4 | Peroxiredoxin 2 | *Brassica rapa* 4928472 | 16 | 3 | 0.05 | 0.07 | 0.24 | 0.15 | 0.16 | | 0.13 | 0.26 | 0.15 | 0.15 | | 0.08 | 0.28 | 0.17 | 0.23 |
| 80 | 35/4.3 | 29/5.2 | Peroxiredoxin | *Phaseolus vulgaris* 11558242 | 30 | 11 | 0.12 | 0.08 | 0.08 | 0.06 | 0.04 | | 0.07 | 0.08 | 0.05 | 0.00 | | 0.04 | 0.10 | 0.04 | 0.00 |
| A55 | 28/5.6 | 25/5.9 | Glutathione S-transferase | *Silene vulgaris* 417093 | 9 | 2 | 0.09 | 0.09 | 0.37 | 0.17 | 0.11 | | 0.17 | 0.15 | 0.19 | 0.20 | | 0.23 | 0.16 | 0.10 | 0.39 |
| G63 | 47/5.7 | 39/6.3 | Peroxidase | *Euphorbia characias* 56385009 | 10 | 3 | 0.39 | 0.45 | 0.22 | 0.07 | 0.09 | | 0.38 | 0.28 | 0.12 | 0.12 | | 0.15 | 0.12 | 0.51 | 0.00 |
| Protein synthesis | | |  |  |  |  |  | | | | | | | | | | | | | | |
| A12 | 41/5.1 | 24/5.1 | Elongation factor 2 (EF-2) | *Triticum monococcum* 58500286 | 29 | 7 | 0.05 | 0.04 | 0.01 | 0.00 | 0.11 | | 0.05 | 0.05 | 0.04 | 0.07 | | 0.04 | 0.00 | 0.05 | 0.06 |
| G41 | 92/6.3 | 95/5.9 | Elongation factor 2 (EF-2) | *Beta vulgaris* 6015065 | 20 | 16 | 0.21 | 0.25 | 0.20 | 0.22 | 0.08 | | 0.21 | 0.18 | 0.14 | 0.17 | | 0.26 | 0.17 | 0.14 | 0.19 |
| A25 | 35/5.2 | 35/5.1 | 60S acidic ribosomal protein P0 | *Lupinus luteus* 1710585 | 13 | 4 | 0.09 | 0.36 | 0.02 | 0.10 | 0.05 | | 0.32 | 0.02 | 0.07 | 0.01 | | 0.16 | 0.18 | 0.19 | 0.01 |
| Development | | |  |  |  |  |  | | | | | | | | | | | | | | |
| A56 | 21/5.5 | 11/6.8 | Em protein | *Robinia pseudoacacia* 1754979 | 13 | 1 | 0.22 | 0.17 | 0.20 | 0.08 | 0.16 | | 0.18 | 0.22 | 0.10 | 0.10 | | 0.19 | 0.13 | 0.14 | 0.20 |
| AG23 | 17/4.7 | 8/5.7 | Lemmi9 | *S. lycopersicum* 1171572 | 17 | 1 | 1.46 | 0.14 | 2.08 | 0.27 | 0.97 | | 0.13 | 1.53 | 1.47 | 1.65 | | 0.68 | 1.38 | 1.14 | 2.14 |
| Cell structure | | |  |  |  |  |  | | | | | | | | | | | | | | |
| 32 | 60/4.9 | 50/5.0 | Alpha-tubulin | [*Anemia phyllitidis* 464840](http://212.87.29.243/mascot/cgi/protein_view.pl?file=../data/20061220/F029087.dat&hit=gi|464840&px=1&protscore=85.93&_mudpit=1&_ignoreionsscorebelow=50) | 4 | 1 | 0.03 | 0.06 | 0.05 | 0.00 | 0.02 | | 0.04 | 0.02 | 0.01 | 0.00 | | 0.01 | 0.03 | 0.02 | 0.00 |
| 35 | 35/4.8 | 51/4.7 | Beta-tubulin | [*Setaria viridis* 51988178](http://212.87.29.243/mascot/cgi/protein_view.pl?file=../data/20060724/F023227.dat&hit=gi|51988178&px=1&protscore=1000.4837037037&_mudpit=1&_ignoreionsscorebelow=35) | 57 | 33 | 0.19 | 0.11 | 0.14 | 0.11 | 0.06 | | 0.12 | 0.05 | 0.08 | 0.05 | | 0.10 | 0.09 | 0.09 | 0.09 |
| Miscellaneous | | |  |  |  |  |  | | | | | | | | | | | | | | |
| A47 | 109/4.7 | 20/10.1 | Hypothetical protein | [*O. sativa* 51091732](http://212.87.29.243/mascot/cgi/protein_view.pl?file=../data/20061220/F029068.dat&hit=gi|51091732&px=1&protscore=62.28&_mudpit=1&_ignoreionsscorebelow=50) | 4 | 1 | 0.43 | 0.22 | 0.28 | 0.00 | 0.07 | | 0.13 | 0.28 | 0.12 | 0.34 | | 0.33 | 0.25 | 0.15 | 0.21 |
| A49 | 40/5.1 | 41/5.6 | Hypothetical protein | [*A. thaliana* 62321134](http://proteom.pl/mascot/cgi/protein_view.pl?file=../data/20060928/F026132.dat&hit=gi|62321134&px=1&protscore=357.24690887108&_mudpit=99999999&_ignoreionsscorebelow=40) | 11 | 4 | 0.11 | 0.03 | 0.05 | 0.09 | 0.06 | | 0.04 | 0.04 | 0.04 | 0.12 | | 0.08 | 0.05 | 0.04 | 0.04 |

The proteins identified by ESI MS/MS were classified according to Bevan *et al.* [19] and included an assigned spot number (Fig. 2). *^a^* The letter A indicates that protein volume variation was dependent on ABA, the letter G, that it was dependent on GA. No letter indicates that the protein varied only during stratification in water. *^b^* % Cov.: percentage of sequence coverage. *^c^* Peptides: number of identified peptides. *^d^* Mean value of the three spot volumes at selected analysed weeks of stratification. Dry: dry dormant seeds, g: germinated seeds. Data were obtained from three biological replicates. - significantly different at p<0.05, - p<0.01,

- p<0.001.
